# MR augmented cardiopulmonary exercise testing - a novel method of assessing cardiovascular function

**DOI:** 10.1186/1532-429X-17-S1-Q2

**Published:** 2015-02-03

**Authors:** Emmanuel O Ako, Nathaniel Barber, Grzegorz T Kowalik, Jennifer Steeden, Vivek Muthurangu

**Affiliations:** 1 The Hatter Cardiovascular Institute,, University College London, London, UK; 2 UCL Institute of Cardiovascular Science & Great Ormond Street Hospital for Children, London, UK

## Background

Reduced exercise capacity is a common feature of many cardiovascular diseases. Quantitative assessment of exercise capacity is usually achieved by measuring peak oxygen consumption (VO_2_). However, measuring peak VO_2_ alone neglects the different components of reduced exercise capacity: namely reduced cardiac output (CO) and oxygen extraction (ΔcO_2_). A better approach would be to simultaneously measure VO_2_ and CO and then calculate ΔcO_2_. This could be achieved using MR augmented cardio-pulmonary exercise testing (MR-CPET)

The aims of this study were to demonstrate: 1) MR-CPET is feasible and well tolerated, 2) peak VO_2_ in the MR scanner correlates with conventional peak VO_2_, and 3) variation in peak VO_2_ is related to both peak CO and peak oxygen extraction (ΔcO_2_) as calculated by the Fick equation.

## Methods

17 healthy volunteers (21-55 years) underwent MR-CPET. Exercise was performed on MR-compatible ergometer (Lode, Groningen, The Netherlands) and VO_2_ was assessed using a commercial respiratory gas analyzer (Ultima, MedGraphics, St. Paul, USA) with a modified sampling tube that was MR compatible. Aortic flow was continuously measured using a previously validated UNFOLD-SENSE spiral PCMR sequence. Images were reconstructed using a graphical processing units card and analyzed using an in-house plug-ins for OsiriX software. Conventional CPET was also performed within 2 weeks of MR-CPET.

For both test, participants were asked to rate i) concern ii) comfort and iii) perceived helplessness.

## Results

15 out of 17 volunteers completed exercise; exclusions were due to claustrophobia (n=1) and inability to master exercise technique (n=1). Reported concern and discomfort was higher with MR-CPET, although still within acceptable limits.

**Figure 1:**
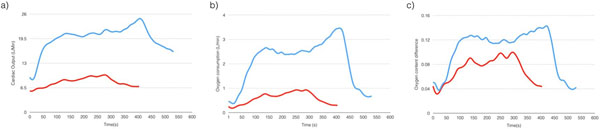


Peak VO_2_, peak VCO_2_ and VE showed strong correlation between conventional CPET and MR-CPET: VO_2_ peak (r=0.94, p<0.001); VCO_2_ (r=0.87, p<0.001); VE (r=0.88, p<0.001). Resting and peak values VO_2_, CO, HR, SV and ΔcO_2_ are shown in table 1. Multiple linear regression analysis demonstrated that both peak CO and ΔcO_2_ were independent predictors of peak VO_2_ measured during MR-CPET (beta=0.73 and 0.38 respectively, p<0.0001) and conventional CPET (beta=0.78, 0.28 respectively, p<0.0001). Representative VO_2_, CO and ΔcO_2_ are shown in figure 1.

**Table 1:** Resting and peak values during MR-CPET

	Resting	Peak	
Variable	Mean (SD)	Mean (SD)	p

Heart Rate (bpm)	73.70 (27.18)	132.08 (15.40)	<0.0001

Stroke Volume (ml/beat)	106.63 (30.91)	110.36 (27.76)	0.42

Cardiac Output (l/min)	7.59 (4.67)	14.36 (1.73)	<0.0001

VO2 (l/min)	0.25 (0.77)	1.56 (0.07)	<0.0001

ΔO2content	0.03 (0.02)	0.11 (0.01)	<0.0001

## Conclusions

MR-CPET is feasible, well tolerated and demonstrates physiology not apparent with conventional CPET. In this study, we have shown that MR-CPET allows assessment of the differing contributions of CO and ΔcO_2_ to variation in peak VO_2_. We believe that will be useful in understanding to origin of reduced exercise capacity in cardiac disease.

## Funding

British Heart Foundation, Great Ormond Street Children's Charity.

